# Suppression of p75 Neurotrophin Receptor Surface Expression with Intrabodies Influences Bcl-xL mRNA Expression and Neurite Outgrowth in PC12 Cells

**DOI:** 10.1371/journal.pone.0030684

**Published:** 2012-01-24

**Authors:** Congcong Zhang, Saskia Helmsing, Marta Zagrebelsky, Thomas Schirrmann, Andrea L. J. Marschall, Manuela Schüngel, Martin Korte, Michael Hust, Stefan Dübel

**Affiliations:** 1 Institute for Biochemistry and Biotechnology, Technische Universität Braunschweig, Braunschweig, Germany; 2 Zoological Institute, Technische Universität Braunschweig, Braunschweig, Germany; 3 Integrated Research and Treatment Center Transplantation, Medical School of Hannover, Hannover, Germany; Carl-Gustav Carus Technical University-Dresden, Germany

## Abstract

**Background:**

Although p75 neurotrophin receptor (p75NTR) is the first neurotrophin receptor isolated, its diverse physiological functions and signaling have remained elusive for many years. Loss-of-function phenotypic analyses for p75NTR were mainly focused at the genetic level; however these approaches were impacted by off-target effect, insufficient stability, unspecific stress response or alternative active splicing products. In this study, p75NTR surface expression was suppressed for the first time at the protein level by endoplasmic reticulum (ER) retained intrabodies.

**Results:**

Three monoclonal recombinant antibody fragments (scFv) with affinities in the low nanomolar range to murine p75NTR were isolated by antibody phage display. To suppress p75NTR cell surface expression, the encoding genes of these scFvs extended by the ER retention peptide KDEL were transiently transfected into the neuron-like rat pheochromocytoma cell line PC12 and the mouse neuroblastoma x mouse spinal cord hybrid cell line NSC19. The ER retained intrabody construct, SH325-G7-KDEL, mediated a downregulation of p75NTR cell surface expression as shown by flow cytometry. This effect was maintained over a period of at least eight days without activating an unfolded protein response (UPR). Moreover, the ER retention of p75NTR resulted in downregulation of mRNA levels of the anti-apoptotic protein Bcl-xL as well as in strong inhibition of NGF-induced neurite outgrowth in PC12 cells.

**Conclusion:**

The ER retained intrabody SH325-G7-KDEL not only induces phenotypic knockdown of this p75NTR but also p75NTR-associated cellular responses in PC12 cells.

## Introduction

Neurotrophins have been known as the critical factors in development and functioning of the nervous system [1]. It has been demonstrated that neurotrophins exert their effects such as proliferation, differentiation, survival and apoptosis by binding to two types of surface receptors, the tyrosine kinase receptor (Trk) family and the p75 neurotrophin receptor (p75NTR). In contrast to Trk receptors, p75NTR binds to all neurotrophins without selectivity. P75NTR normally collaborate with many different protein partners [2]. Although p75NTR is the first neurotrophin receptor identified, its precise physiological role is still conflicting to date [3]. P75NTR is usually considered as a proapoptotic receptor; however, it has also been found that p75NTR enhanced the survival of neurons in the presence of Trk receptors [4]. A survival response was also reported in rat Schwann cells, in which nuclear factor kappa B (NFκB) was activated via NGF-induced p75NTR signaling pathway [5]. It has been reported that Trk receptors are essential for neurite outgrowth and p75NTR was shown to negatively alter dendrite complexity and spine density in hippocampal pyramidal neurons [6]. Nevertheless, it has also been demonstrated that applying an anti-p75NTR antibody, MC192, can inhibit NGF-dependent neurite extensions of hippocampal neurons [7] and sensory neurons [8].

Many approaches have been applied to investigate p75NTR *in vivo* and *in vitro*, such as gene-targeted knockout mice, antisense RNA, and siRNA, but none of them performs modulations at the post-translational level. Endoplasmic reticulum (ER) retained intrabodies can specifically trap target proteins within the ER based on an ER retention signal at the C-terminus (for review see Böldicke, 2007) [9]. This technology takes advantage of the naturally occurring mechanism of ER-resident protein retrotranslocation in mammalian cells. KDEL, a conserved carboxy-terminal tetrapeptide, is the most common ER retention signal used for this purpose. In contrast to gene-silencing knockdown techniques, ER retained intrabodies offer the possibility to neutralize the functions of proteins at the post-translational level. Besides the high affinity and specificity provided by an antibody moiety, ER retained intrabodies are expressed very stably in mammalian cells compared to siRNAs [10] or aptamers [11]. The functional knockdown of vascular cell adhesion molecule 1 (VCAM-1) has been recently demonstrated by using an intrabody [12].

**Figure 1:**
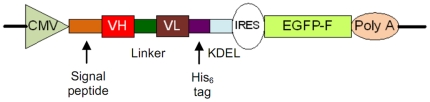
Schematic representation of the bicistronic knockdown vector. CMV: cytomegalovirus immediate-early promoter; VH: variable domain of heavy chain; VL: variable domain of light chain; KDEL: C-terminal ER retention signal; IRES: internal ribosomal entry site; EGFP-F: farnesylated enhanced green fluorescent protein; PolyA: BGH polyadenylation sequence.

In this study, recombinant antibodies specific to p75NTR were selected by antibody phage display technology (for review see Dübel et al. 2010) [13]. Based on screening high diversity ‘single-pot’ antibody gene libraries, monoclonal antibody fragments with high specificities and affinities can be selected rapidly [14], [15], [16]. The whole screening procedure, called panning, is done completely *in vitro* and offers speed and properties difficult to obtain with hybridoma technique [17], [18]. For detailed analysis of intrabody-induced knockdown, we constructed a novel bicistronic knockdown vector, encoding both the intrabody and farnesylated fluorescent reporter protein (Fig. 1). The effect of anti-p75NTR intrabody production on stress response, Bcl-xL mRNA expression, and NGF-induced neurite outgrowth of PC12 cells were analyzed.

## Results

### Selection of recombinant scFvs against the extracellular domain of p75NTR by antibody phage display

Antibody phage display technology was applied to isolate scFvs against the extracellular domain of p75NTR. The antigen p75NTRex-Fc protein was prepared by fusing mouse p75NTR extracellular domain DNA (a kind gift from RZPD, Germany) with the human IgG1 Fc domain gene. After transient production in HEK 293T cells, the p75NTRex-Fc fusion protein was purified by protein A affinity chromatography before being used for panning with naïve human antibody gene libraries HAL4 (Kappa) and HAL7 (Lambda) [16]. In order to avoid the selection of scFvs against human IgG1 Fc portion, N protein standard SL (Dade Benring, Germany), a human serum standard containing human IgG, was used as soluble competitor during the panning procedure. After three rounds of panning and selection, individual clones were isolated. Soluble monoclonal scFvs were produced in *E. coli* cultured in microtiter plates [19] and assessed for their binding abilities to the immobilized p75NTRex-Fc fusion protein by ELISA (data not shown). Three unique p75NTR-specific scFvs (SH325-A11, SH325-B6, and SH325-G7) were finally identified after DNA sequencing. Their sequences were analyzed based on the integrative database of germline variable genes from the immunoglobulin loci of human (VBASE2, http://www.vbase2.org/) [20]. The VH of all three antibodies were derived from a VH3 germline gene. The light chains were of lambda type in all three cases with the V genes from different germline genes (Table 1).

**Table 1:** P75NTR-specific scFvs isolated by phage display.

Antibody	VH			VL	
clone	V	D	J	V	J
SH325-A11	IGHV3-23*01	IGHD6-13*01	IGHJ4*02	IGLV6	IGLJ3*02
SH325-B6	IGHV3	IGHD6-19*01	IGHJ4*02	IGLV3-21*02	IGLJ3*01
SH325-G7	IGHV3	IGHD2-15*01	IGHJ3*02	IGLV1-44*01	IGLJ3*02

The names of gene segments are as given in VBASE2. V: variable gene; D: diversity gene segment; J: joining gene segment.

### Characterization of the p75NTR-specific scFvs

The p75NTR-specific scFvs were produced by periplasmic expression in *E. coli* and purified by immobilized metal affinity chromatography (IMAC). The binding kinetics of the p75NTR-specific scFvs to the solid phase immobilized p75NTRex-Fc fusion protein was determined by surface plasmon resonance (SPR) spectroscopy (Biacore). Recombinant rat TrkAex-Fc fusion protein was used as a negative control. The measured affinities were in a low nanomolar range (3.9–39 nM, see Table 2).

**Table 2:** Binding kinetics of the p75NTR-specific scFv as determined by surface plasmon resonance.

	K_a_ (1/M⋅s)	k_d_ (1/s)	Rmax (RU)	*X^2^*	K_D_ (M)
SH325-A11	2.44×10^5^	1.57×10^−3^	156	2.31	6.5×10^−9^
SH325-B6	4.45×10^5^	1.74×10^−3^	162	4.43	3.9×10^−9^
SH325-G7	1.08×10^5^	4.22×10^−3^	180	0.24	3.9×10^−8^

**Figure 2:**
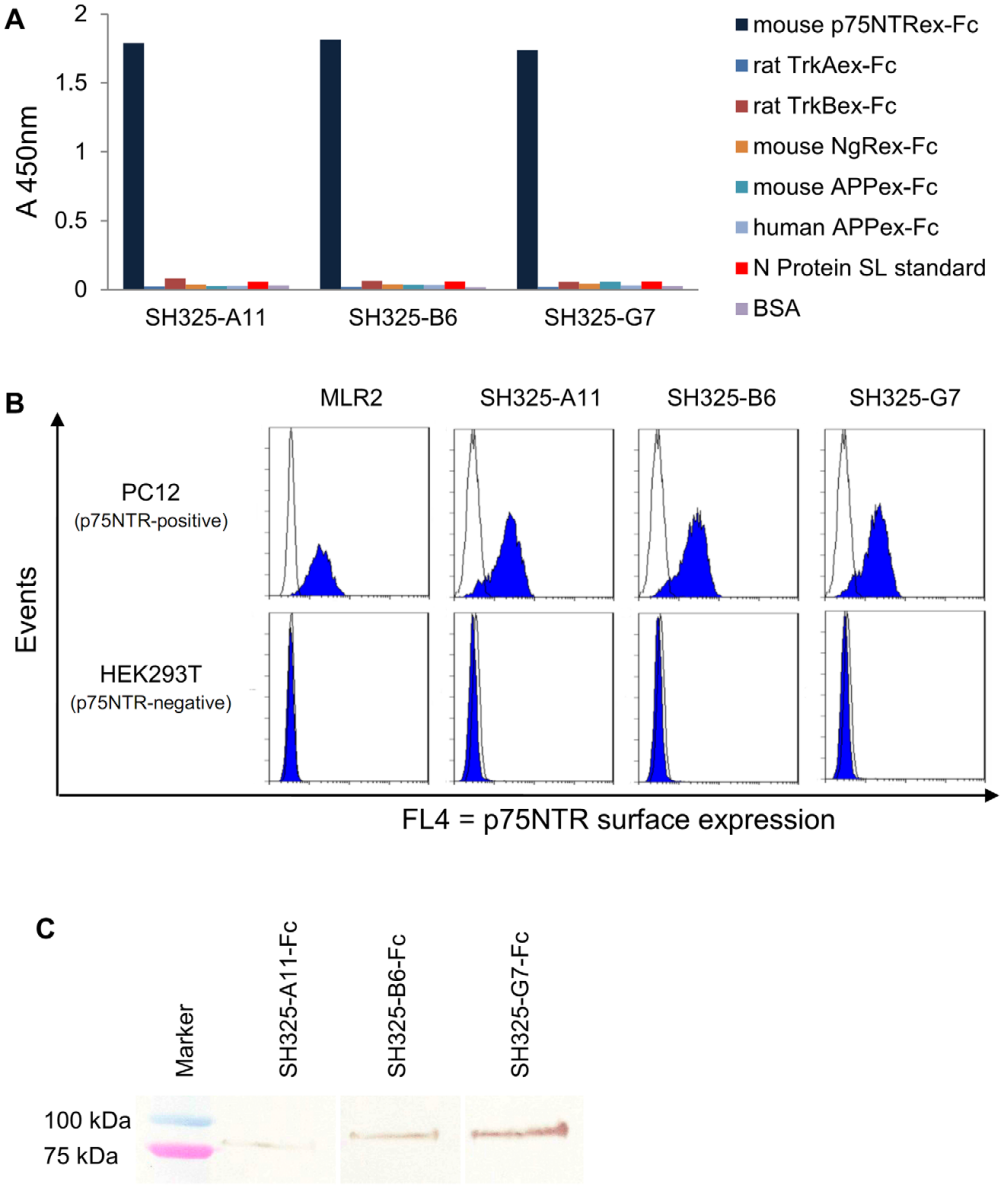
Characteration of the p75NTR-specific antibodies. (A) Antigen binding ELISA. 100 ng of mouse p75NTRex-Fc (shown in dark blue), rat TrkAex-Fc, rat TrkBex-Fc, mouse NgRex-Fc, mouse or human APPex-Fc, N protein SL standard or BSA were immobilized in the plate for each well. 250 ng of each scFv was added after the antigen-coated plates were blocked with FCS for 1.5 hr. Bound scFvs were detected using anti myc-tag mAb (1∶500) and goat anti-mouse IgG HRP conjugated (1∶5,000). (B) The scFvs specifically recognize native p75NTR on PC12 cell surfaces. PC12 or HEK293T cells were stained with 250 ng of the p75NTR-specific scFvs (SH325-A11, SH325-B6, SH325-G7). Bound scFvs were detected by mouse anti-His_6_ mAb (1∶100) followed by goat anti-mouse IgG F(ab′)^2^ fragment APC conjugated (1∶200). The p75NTR surface expression on PC12 cells was determined by staining PC12 or HEK293T cells with mouse anti-p75NTR mAb (MLR2, 1∶200) followed by goat anti-mouse IgG F(ab′)^2^ fragment APC conjugated (1∶200). The blue histograms represent the mouse anti-p75NTR mAb (MLR2) or the p75NTR-specific scFvs staining PC12 cells (upper row) and HEK 293T cells (lower row). The white histograms represent the controls stained with goat anti-mouse IgG F(ab′)^2^ fragment APC conjugated alone (MLR2 line) or *α*phOx scFv (other lines) followed by mouse anti-His_6_ mAb and goat anti-mouse IgG F(ab′)^2^ fragment APC conjugated. (**C**) Detection of denatured antigen (p75NTRex-mFc) by the p75NTR-specific recombinant antibodies in immunoblot. 250 ng of p75NTRex-mFc was denatured and blotted on a PVDF membrane. 1 µg of each p75NTR-specific recombinant antibody was used to stain the membrane for 1.5 hr. The bound antibodies were detected by goat anti-human IgG Fc antiserum AP conjugated (1∶2,000) for 1 hr at RT.

Specificity of the p75NTR-specific scFvs was assayed by antigen ELISA to check for cross-reactivity with other related neuronal receptors which may have antagonistic functions to p75NTR [3]. As shown in Figure 2A, all scFvs specifically bound to the p75NTRex-Fc fusion protein but not to other Fc fusion proteins as rat TrkA (TrkAex-Fc), rat TrkB (TrkBex-Fc), mouse Nogo receptor (NgRex-Fc), mouse and human amyloid precursor protein (APPex-Fc). The binding of these scFvs to native antigen on living cells was verified by flow cytometric analysis (Fig. 2B). Since high homology is shown between mouse and rat p75NTR [21], rat pheochromocytoma PC12 cells which are neuron-like cells endogenously expressing p75NTR was used [22]. The p75NTR expression on the PC12 cell surface was monitored by using the commercial mouse anti-p75NTR mAb (MLR2). HEK 293T cells were used as p75NTR negative control cells. A scFv against the hapten 2-phenyloxazoline-5-one [23] was used as the negative control antibody. All the p75NTR-specific scFvs specifically bound to the PC12 cells, while none of them showed binding to the HEK 293T cells.

The interaction between the p75NTR-specific recombinant antibodies and p75NTR extracellular domain was also analyzed by Western blotting (Fig. 2C). 250 ng of p75NTRex-mFc, prepared by fusing the extracellular domain DNA of mouse p75NTR with mouse IgG1 Fc domain gene, was denatured and blotted on a PVDF membrane. The denatured antigen was incubated with three p75NTR-specific scFv human Fc fusion recombinant antibodies (SH325-A11-Fc, SH325-B6-Fc, and SH325-G7-Fc), respectively. Compared to SH325-G7-Fc, SH325-A11-Fc and SH325-B6-Fc showed much weaker signals in immunoblot for detecting the denatured p75NTR.

### Mouse anti-p75NTR mAb (MLR2) and the p75NTR-specific scFvs bind to different, non-overlapping epitopes

**Figure 3:**
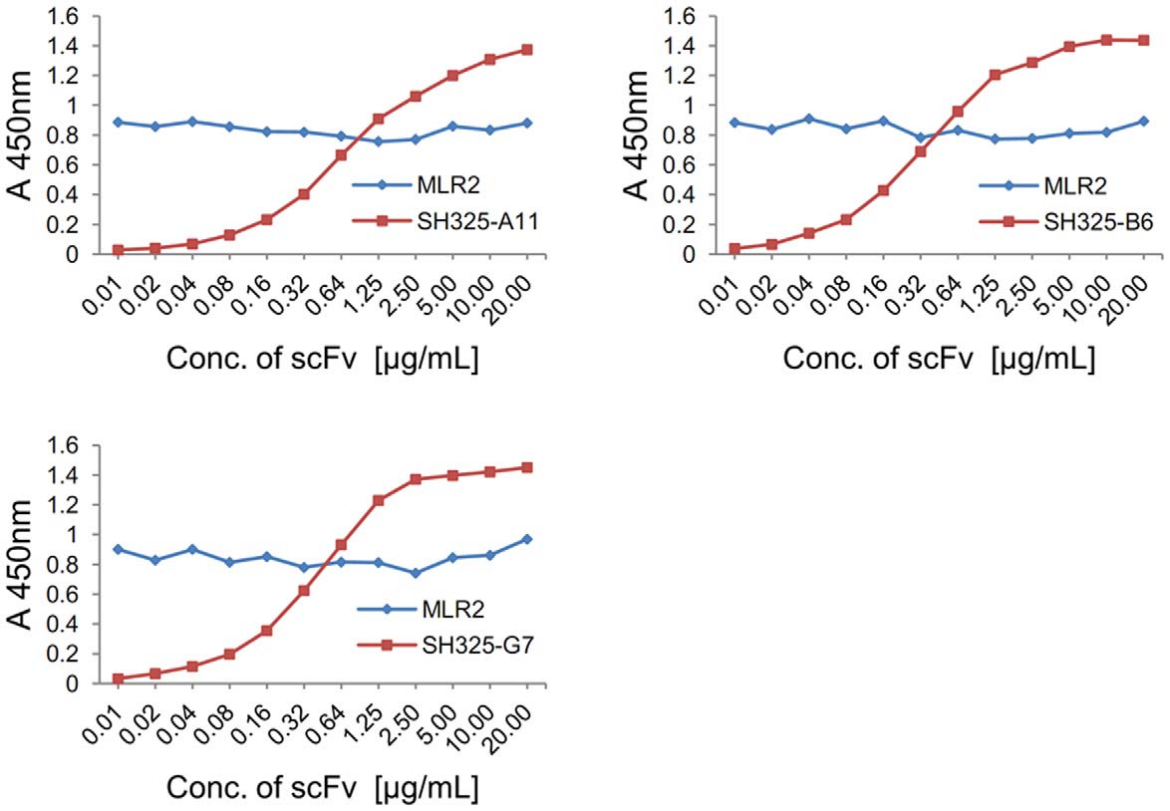
Competition ELISA for epitope overlapping. 100 ng of mouse p75NTRex-Fc fusion protein was immobilized in plates for each well. Serial diluted p75NTR-specific scFvs were added and incubated for 1.5 hr at 37°C. After 3× washing with PBST, the plates were incubated with mouse anti-p75NTR mAb (MLR2, 1∶5,000) for 1.5 hr at 37°C. The plate was washed 3× with PBST. The scFvs and mouse anti-p75NTR mAb (MLR2) were detected by mouse anti-His_5_ mAb HRP conjugated (1∶5,000) and goat anti-mouse IgG HRP conjugated (1∶5,000) in corresponding plates.

To be able to detect changes in surface expression of p75NTR in the presence of intrabodies, it must be assured that the intrabodies do not interfere with the binding of the detection antibodies. To check this, a consecutive competition ELISA was performed with the p75NTR-specific scFvs and the mouse anti-p75NTR mAb (MLR2), which was to be used to analyze the p75NTR surface expression (Fig. 3). Signal intensities of three p75NTR-specific scFvs increased in a concentration dependent way (Fig. 3 red lines). No significant change in signal intensities was observed for the mouse anti-p75NTR mAb (MLR2) in the presence of the different scFv concentrations (Fig. 3, blue lines). This indicates that the binding of mouse anti-p75NTR mAb (MLR2) to p75NTR was not inhibited by the p75NTR-specific scFvs.

### Knockdown of p75NTR surface expression in PC12 cells by p75NTR-specific ER retained intrabodies

**Figure 4:**
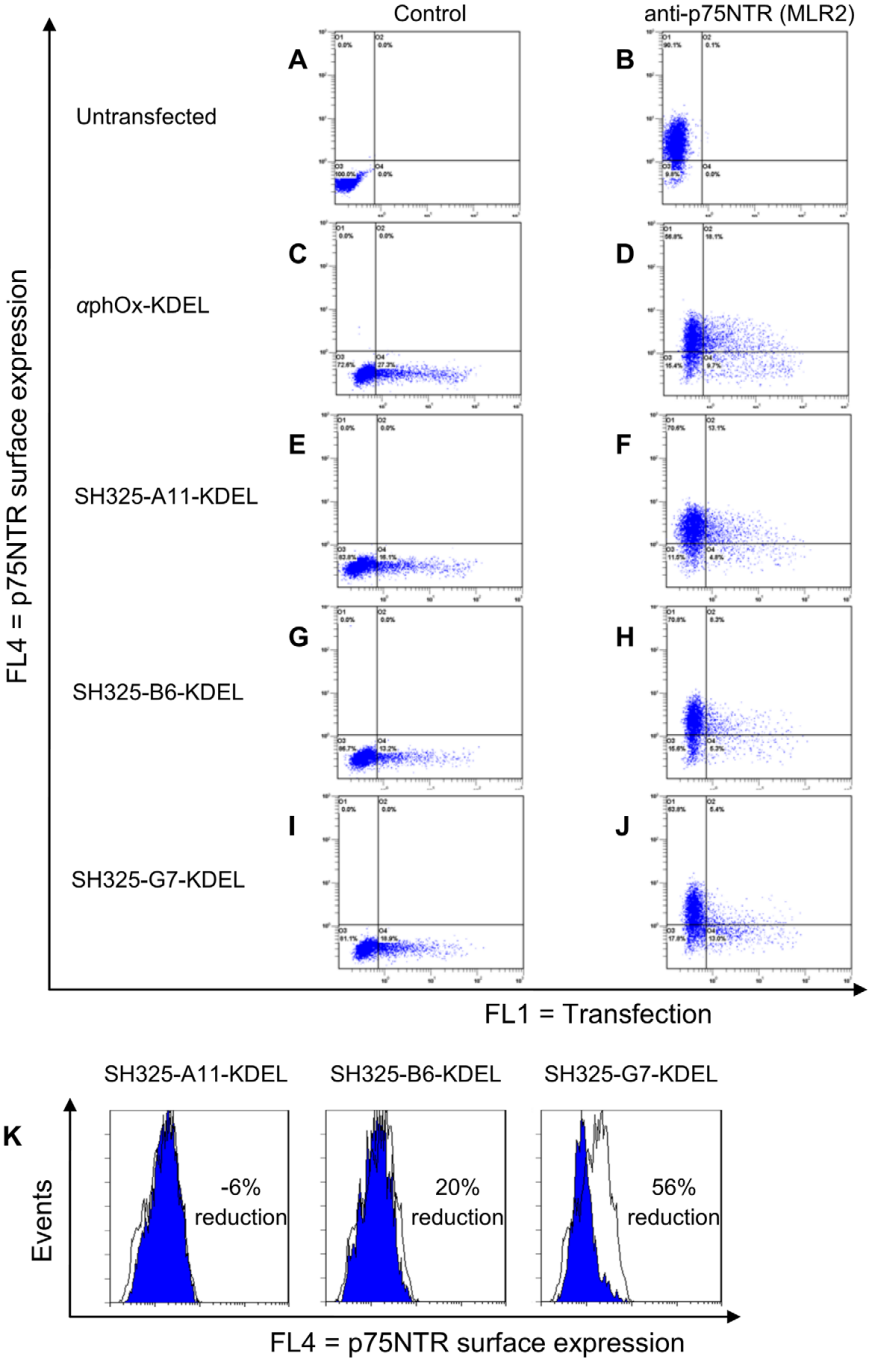
Knockdown of p75NTR surface expression in PC12 cells by the p75NTR-specific ER retained intrabodies. PC12 cells were transiently transfected with the p75NTR-specific ER retained intrabodies or *α*phOx-KDEL. Four days after transfection, the p75NTR surface expressions were determined with mouse anti-p75NTR mAb (MLR2, 1∶200) followed by goat anti-mouse IgG F(ab′)^2^ fragment APC conjugated (1∶200, B, D, F, H, and J). Cells stained with secondary antibody alone were served as control (A, C, E, G, and I). Overlay analysis for the p75NTR surface expression is shown in PC12 cells (K). The blue histograms represent the p75NTR surface expression of the PC12 cells expressing the p75NTR-specific ER retained intrabodies. The white histograms represent the p75NTR surface expression of the PC12 cells expressing the control *α*phOx-KDEL. The efficiency of knockdown p75NTR surface expression is indicated in percent. The picture of SH325-G7-KDEL is also shown in Figure 7 as 4 day time point result.

The gene encoding the p75NTR-specific scFvs were subcloned into the bicistronic knockdown vector to generate ER retained intrabodies (SH325-A11-KDEL, SH325-B6-KDEL and SH325-G7-KDEL). This knockdown vector adds a C-terminal ER retention motif KDEL to the scFv fragments. The first cistron with the ER retained intrabody is controlled by a CMV promoter, while a farnesylated form of EGFP (EGFP-F) was driven as second cistron by a mutant EMCV IRES element in cap-independent mechanism [24]. Because the ER retained intrabody and EGFP-F are simultaneously translated from a single transcript, the intrabody-expressing cells can be easily detected by flow cytometry and fluorescence microscopy. Furthermore, EGFP-F is bound to the inner plasma membrane of cells even after fixation; it thereby facilitates morphological studies, especially of neurons. First, we analyzed the p75NTR surface expression in PC12 cells transiently transfected with the bicistronic knockdown vectors. As a control, the ER retained intrabody *α*phOx-KDEL was used. Four days after transfection, the p75NTR surface expression in PC12 cells was analyzed by flow cytometry (Fig. 4). The untransfected PC12 cells showed p75NTR expression on their cell surfaces (Fig. 4B). Cells transfected with the ER retained intrabodies were identified by co-expression of EGFP-F reporter gene. If the p75NTR surface expression is downregulated by the ER retained intrabodies, the cell population is expected to be shifted from the upper right quadrant to the lower right quadrant. High levels of p75NTR were expressed on the surfaces of the cells transfected with *α*phOx-KDEL control construct (Fig. 4D). No considerable reduction of the p75NTR surface expression was detected in the SH325-A11-KDEL transfected cells (Fig. 4F). In the cells transfected with SH325-B6-KDEL, a moderate decrease of p75NTR surface expression was measured (Fig. 4H). P75NTR surface translocation was remarkably inhibited in the SH325-G7-KDEL transfected cells, resulting in a shift of the transfected cell population from the FL4^+^/FL1^+^ to the FL4^−^/FL1^+^ quadrant (Fig. 4J). Unspecific binding from the detection antibody was excluded by using negative controls stained with the secondary antibody alone (Fig. 4A, 4C, 4E, 4G, and 4I). An overlay analysis was performed between the cells expressing the p75NTR-specific ER retained intrabodies and those expressing the control construct (Fig. 4K). By comparing the mean fluorescence intensities between the cells expressing the p75NTR-specific ER retained intrabody SH325-G7-KDEL and the cells expressing the control *α*phOx-KDEL, the efficiency of knockdown p75NTR surface expression was 56%.

### P75NTR surface expression was downregulated by the p75NTR-specific ER retained intrabodies in NSC19 cells

**Figure 5:**
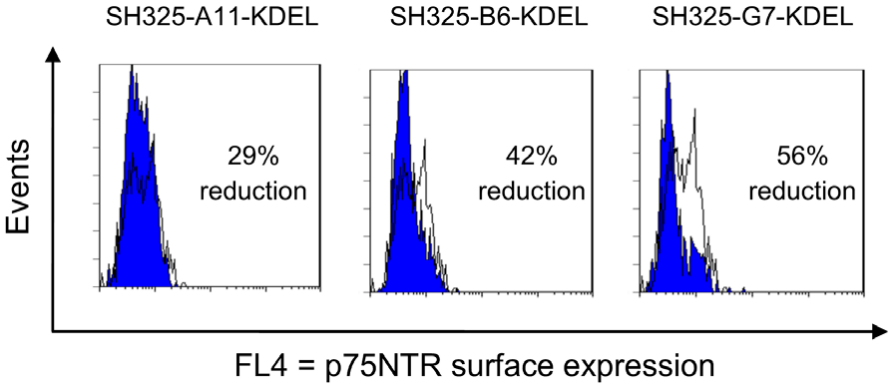
Knockdown of p75NTR surface expression in NSC19 cells by the p75NTR-specific ER retained intrabodies. NSC19 cells were transiently transfected with the p75NTR-specific ER retained intrabodies or the control *α*phOx-KDEL. Four days after transfection, the p75NTR surface expressions were determined with mouse anti-p75NTR mAb (MLR2, 1∶200) followed by goat anti-mouse IgG F(ab′)^2^ fragment APC conjugated (1∶200). The blue histograms represent the p75NTR surface expressions of the NSC19 cells expressing the p75NTR-specific ER retained intrabodies. The white histograms represent the p75NTR surface expressions of the NSC19 cells expressing the control *α*phOx-KDEL. The efficiency of knockdown p75NTR surface expression is indicated in percent.

The effect of p75NTR-specific ER retained intrabodies was also analyzed in mouse NSC19 cells [25]. Four days after transfection with the ER retained intrabodies, the NSC19 cells were stained for p75NTR and subjected to flow cytometric analysis. The efficiency of knockdown p75NTR surface expression was calculated as described above. The effects of the three p75NTR-specific ER retained intrabodies were comparable to the effects observed in PC12 cells (Fig. 5). Again, the most distinctive effect was seen with SH325-G7-KDEL (56% reduction). SH325-A11-KDEL or SH325-B6-KDEL transfected cells showed 29% or 42% reduction on the p75NTR surface expression, respectively.

### Intracellular expression levels of the p75NTR-specific ER retained intrabodies

**Figure 6:**
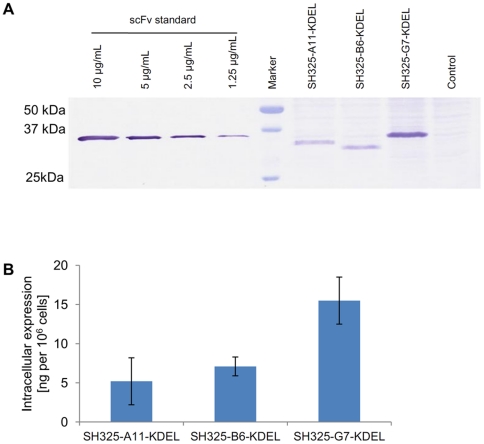
Intracellular expression levels of the p75NTR-specific ER retained intrabodies in PC12 cells. (A) Quantitative determination by immunoblotting. PC12 cells were transiently transfected with the constructs encoding the p75NTR-specific ER retained intrabodies. Four days after transfection, cell extracts were prepared and separated by SDS-PAGE. After immunoblotting, the membrane was incubated with mouse anti-His_5_ mAb (1∶2,000) for 1 hr at RT and followed by goat anti-mouse IgG AP conjugated (1∶5,000) for 1 hr at RT. The control resulted from the cell extracts of untransfected PC12 cells. Intracellular expression levels of the p75NTR-specfic ER-intrabodies were calculated according to the scFv standard. (B) Quantification of intrabody production by densitometric analysis of gels as shown in (A). The error bars represent standard deviations calculated from three independent transfection experiments.

To figure out the reason for the difference among the knockdown efficiencies, the amount of intracellular ER retained intrabodies was analyzed after transient transfection of knockdown vectors encoding the p75NTR-specific ER retained intrabodies into PC12 cells. Transfection efficiencies were determined in different experiments in flow cytometry. Four days after transfection, 2×10^6^ cells from each sample were lysed in 200 µL of lysis buffer. The intracellular expression of the p75NTR-specific ER retained intrabodies was evaluated by immunoblotting and bands corresponding to the calculated molecular masses were identified (Fig. 6A). A negative control resulted from the cell extracts of untransfected PC12 cells. The signal intensities were analyzed by ImageJ software. The intracellular expression level of SH325-G7-KDEL was approximately 2–3 times more than those of SH325-A11-KDEL and SH325-B6-KDEL in transiently transfected PC12 cells (Fig. 6B).

### P75NTR surface expression was suppressed by the p75NTR-specific ER retained intrabody for at least 8 days in PC12 cells

**Figure 7:**
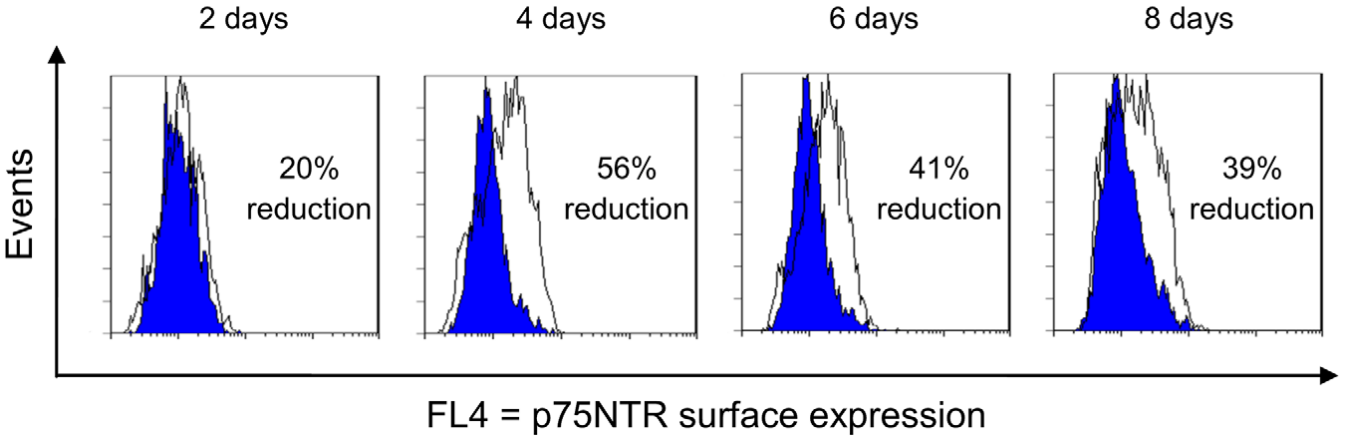
Kinetics of the p75NTR knockdown effect in PC12 cells. PC12 cells were transiently transfected with SH325-G7-KDEL or the control *α*phOx-KDEL and harvested at different time points. The p75NTR surface expression levels were determined with mouse anti-p75NTR mAb (MLR2, 1∶200) followed by goat anti-mouse IgG F(ab′)^2^ fragment APC conjugated (1∶200). The blue histograms represent the p75NTR surface expressions of the PC12 cells expressing SH325-G7-KDEL. The white histograms represent the p75NTR surface expressions of the PC12 cells expressing *α*phOx-KDEL. The efficiency of knockdown p75NTR surface expression is indicated in percent.

The kinetics of the p75NTR-specific knockdown by ER retained intrabodies after transiently transfection in PC12 cells was analyzed over 8 days using the construct of SH325-G7-KDEL (Fig. 7). The effect of p75NTR-specific ER retained intrabody progressively increased from 2 to 4 days after transfection with a maximal knockdown (56% reduction) at day 4 post-transfection. Afterwards, the suppression of p75NTR surface expression slightly decreased to 39% after 8 days.

### P75NTR-specific ER retained intrabody expression does not activate the unfolded protein response (UPR) in PC12 cells

**Figure 8:**
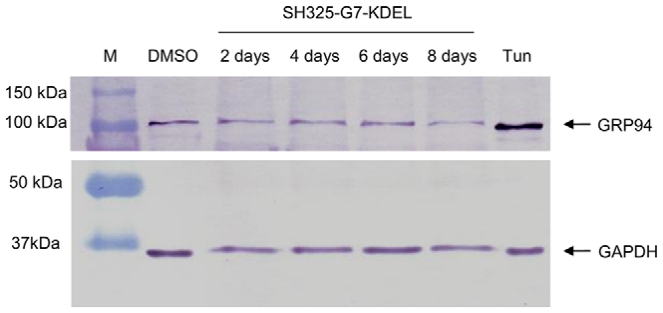
Stress response (UPR) in cells transfected with intrabody construct SH325-G7-KDEL. The SH325-G7-KDEL transfected PC12 cells from different time points (2–8 days) were sorted based on the EGFP-F fluorescence. Cells treated with 20 µg/mL of tunicamycin (Tun) or solvent alone (DMSO) were used as the positive or negative control, respectively. Cell extracts were prepared and blotted on a PVDF membrane. The membrane was incubated with rabbit anti-GRP94 (1∶1,000) and rabbit anti-GAPDH (1∶5,000) for 1 hr at RT. After 3× washing with PBST, the membrane was subsequently incubated with goat anti-rabbit IgG AP conjugated antibody (1∶5,000) for 1 hr at RT. GAPDH served as an internal control to ensure equal protein loading.

To assess the stress imposed to the cells by the of the specific ER retained intrabody productions, UPR was analyzed during the knockdown process up to 8 days. ER resident 94 kDa glucose-regulated protein (GRP94) was used as the reporter protein for ER stress response. Its expression is upregulated due to ER stress [26]. PC12 cells were transiently transfected with SH325-G7-KDEL. Cells from different time points (2–8 days) were sorted by BD FACSAria™ II based on EGFP-F fluorescence to eliminate nontransfected cells. Negative and positive controls were prepared by treating PC12 cells for 24 hr with DMSO alone or 20 µg/mL of tunicamycin, respectively. Tunicamycin is known as an UPR stimulus because it inhibits N-linked glycosylation [27] and influences glycoprotein membrane transport [28]. The expression levels of GRP94 were evaluated by immunoblotting (Fig. 8). Glyceraldehyde 3-phosphate dehydrogenase (GAPDH) served as an internal reference to ensure equal total protein loading and was detected on the same membrane. Intrabody production did not lead to any detectable GRP94 upregulation compared to the DMSO treated negative control. In contrast, tunicamycin treated cells showed a considerable increase of GRP94 expression.

### Downregulation of p75NTR surface expression by the ER retained intrabody reduces Bcl-xL mRNA expression in PC12 cells

**Figure 9:**
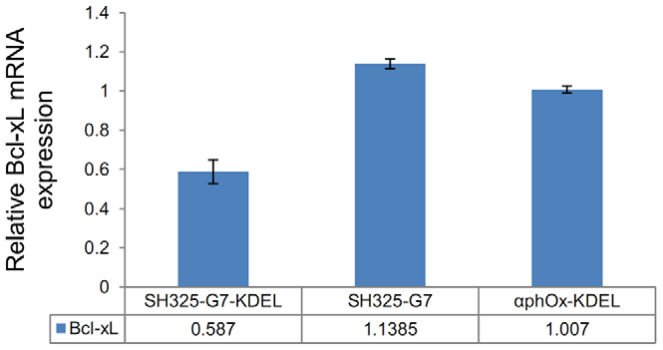
Bcl-xL mRNA expression correlates with p75NTR surface expression knockdown. PC12 cells were transfected with the constructs of SH325-G7-KDLE, or the negative controls SH325-G7 or *α*phOx-KDEL. Six days after transfection, total RNA was extracted. The Bcl-xL mRNA expression, normalized to the housekeeping gene β-actin, was determined by real-time qRT-PCR. These results were then compared to the normalized Bcl-xL expression in untransfected PC12 cells. The error bars represent standard deviations from two independent experiments.

A previous study reported that constitutive Bcl-xL expression is related to p75NTR signaling in PC12 cells [29]. In order to investigate the impact of the downregualtion of p75NTR surface expression by the ER retained intrabody, Bcl-xL mRNA expression was evaluated by real-time qRT-PCR. Six days after transfection, the Bcl-xL mRNA expression of the cells treated with SH325-G7-KDEL construct was only half as high as that of the untransfected PC12 cells (Fig. 9). In contrast, there is no significant difference in Bcl-xL mRNA expression between the cells transfected with control constructs and untransfected cells. Besides *α*phOx-KDEL, another control construct was SH325-G7 which lacks the ER retention sequence KDEL. It is not capable to retain p75NTR within the ER and did not influence Bcl-xL expression. The results suggest that the reduction of Bcl-xL mRNA expression was caused by the suppression of p75NTR surface expression.

### P75NTR plays an important role in regulating NGF-induced neurite outgrowth in PC12 cells

**Figure 10:**
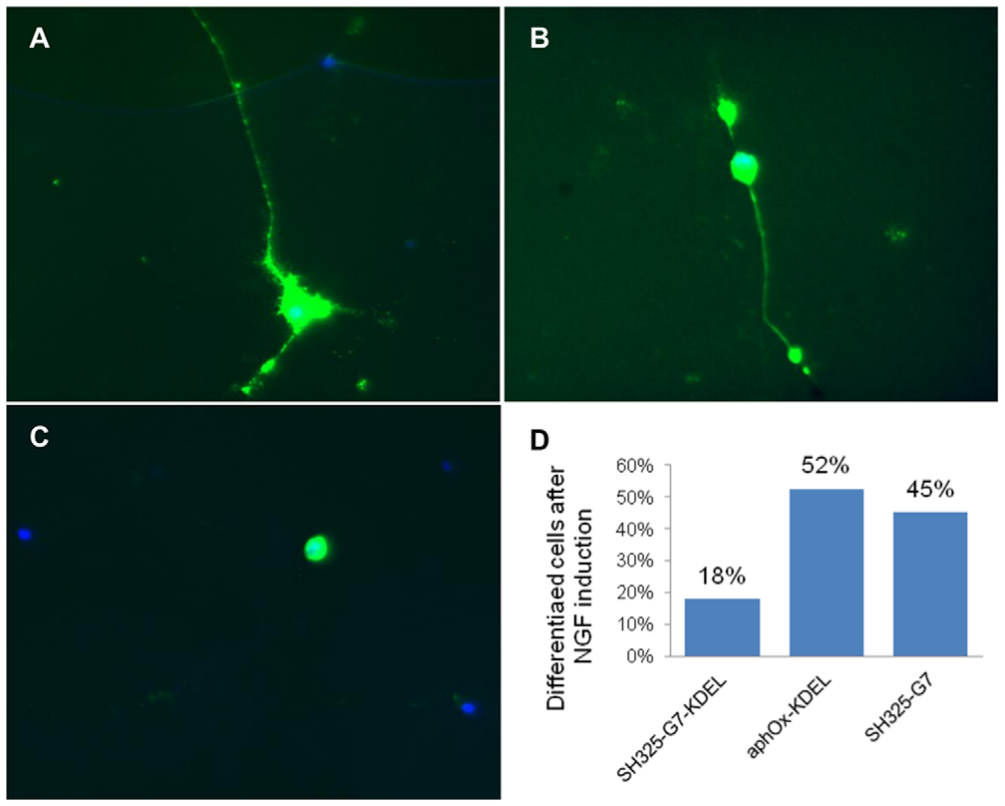
Effect of intrabody expression on the NGF-induced differentiation of PC12 cells. (A) PC12 cells transfected with the *α*phOx-KDEL construct. (B) PC12 cells transfected with the SH325-G7 construct. (C) PC12 cells transfected with SH325-G7-KDEL construct. (D) Percentage of differentiated PC12 cells in the cells transfected with different constructs (about 100 cells counted for each sample).

Further investigation was performed in PC12 cells to study the influence of p75NTR surface knockdown on NGF-induced neurite outgrowth. Cells were transfected with SH325-G7-KDEL, or the controls SH325-G7 or *α*phOx-KDEL. Two days after transfection, the cells were induced with 200 ng/mL NGF for four days. Photographs of transfected cells, as indicated by EGFP-F expression, were collected from each sample under the microscope (Fig. 10). Approximately 100 transfected cells were counted from each sample. The transfection of PC12 cells with SH325-G7-KDEL strongly inhibited the NGF-induced neurite outgrowth (Fig. 10C). In contrast, the PC12 cells transfected with the controls SH325-G7 and *α*phOx-KDEL tended to extend long neurites in the presence of NGF (Fig. 10A, 10B). Quantification revealed that 50% of the cells transfected with the control constructs extended neurites after NGF treatment, whereas only 18% extended neurites after transfected with the SH325-G7-KDEL construct (Fig. 10D).

## Discussion

P75NTR belongs to the tumor necrosis factor receptor (TNFR) superfamily and plays a critical role in almost all aspects of the function of vertebrate neurons. P75NTR has been reported to affect neuronal survival, apoptosis, neurogensis, synaptic plasticity, and long-term depression in ligand-dependent and -independent mechanisms [30], [31], [32], [33]. P75NTR is also known to be linked to many human diseases, including schizophrenia, cerebral edema, neural crest tumors, stroke, Alzheimer's disease and several hereditary neurodegenerative disorders like ataxia-talangectasia and spinocerebella ataxias [34]. So far, most functional studies on p75NTR focused at the genetic level. Gene knockout p75NTR^−/−^ mice are important animal models to study the *in vivo* role of p75NTR. However, gene-targeted knockout is a time-consuming work on the one hand, and alternative splicing products, on the other hand, have been reported in both p75NTR^exonIII^ and p75NTR^exonIV^ knockout phenotypes [35], [36]. These alternative splicing products contain the intact transmembrane and intracellular domain of p75NTR that are suggested to have proapoptotic effects rather than to be only splicing products without any function [37], [38]. RNAi is another promising technique which was frequently used to downregulate p75NTR expression. But RNAi induces unexpected off-target effects on gene expression and the possibility of interferon response induction in cells, which nonspecifically suppresses protein translation [10], [39], [40], [41]. In addition, the general short half-life of siRNA prevents long period studies or therapeutic application [42]. In contrast, ER retained intrabody knockdown technology has not only the advantages of an antibody like high specificity, affinity and stability, but it can also efficiently inhibit the translocation of specific secretory or surface molecules from the ER to the cell surface [9], [12], [43]. In this study, we present a phenotypic knockdown of p75NTR at the post-translational level by using ER retained intrabodies for the first time.

Three scFvs to the extracellular domain of mouse and rat p75NTR, SH325-A11, SH325-B6 and SH325-G7, with high monovalent affinities (3.9 to 39 nM) were selected from the naïve human antibody gene libraries HAL4/7 [16] and tested for intrabody-induced knockdowns. The calculated knockdown never was 100%, but this may be explained at least in part by the overall very low initial expression level of p75NTR on the cells, not allowing baseline separation of the histograms. Interestingly, the most efficient downregulation of surface p75NTR in PC12 and NSC19 cells was achieved with the construct SH325-G7, which has the lowest affinity among the three isolated p75NTR-specific scFvs. In transiently transfected PC12 cells, 2–3 times more SH325-G7-KDEL was produced compared to SH325-A11-KDEL and SH325-B6-KDEL; therefore, the intrabody expression level seems to be one of the pivotal factors impacting ER retention of the target protein. Although increased affinity or avidity also seems important to improve the effect of ER retained intrabodies, higher affinities of the antibodies did surprisingly not increase knockdown efficiency in this study. It should not to be forgotten that different scFvs may recognize different p75NTR epitopes which are not accessible in the same way in the ER, or that folding may not be completed [44]. In respect of the latter, we observed on the immunoblot analysis that SH325-G7 was the best binder for denatured (i.e. unfolded) p75NTR. Therefore, it is possible that antibodies with high affinity to linear epitopes may mediate higher intrabody-induced knockdown efficiency. If so, this effect can be expected to strongly depend on the individual antigen, but is surely worth further evaluation in future intrabody studies.

The p75NTR downregulation by the intrabody SH325-G7-KDEL construct was time dependent and long lasting, with a maximum at day 4 post-transfection and remaining relatively stable for at least 8 days. UPR, a stress response described to be caused by strong protein overexpression in the ER [45], [46], was not observed during this time period. Since UPR was activated neither by the transient transfection procedure nor by the intrabody production, it can be assumed that general protein synthesis was not halted and the ER stress-induced apoptosis was not initiated in the cells expressing the ER retained intrabody, and unspecific off-target effects seem to be negligible [47], [48].

It has been demonstrated that p75NTR can mediate proapoptotic or prosurvival signaling depending on the molecular context [3], [49], [50]. In the present study, we observed that the Bcl-xL mRNA expression was reduced by downregulation of p75NTR cell surface expression. This result is in agreement with a previous study, where constitutive Bcl-xL mRNA expression level was decreased in PC12 cells after blocking cell surface p75NTR by an anti-p75NTR antibody [29]. It might be caused by the p75NTR-dependent PI3-kinase/Akt survival pathway activation [51], which is independent to the TrkA signaling and has been proved to upregulate Bcl-x gene expression [52]. These results suggest that p75NTR surface expression is required for constitutive Bcl-xL expression and may provide support in survival signaling. Furthermore, we found that the neurite outgrowths were substantially repressed by inhibiting p75NTR surface expression in PC12 cell. This repression was not a result of transfection procedures or the additional overexpression of a scFv fragment, since it was not observed with the control antibodies. It is known that p75NTR is heavily colocalized with TrkA on the cell surface and enhances the affinity and selectivity of TrkA for NGF binding [53], [54]. The direct association or indirect communication between p75NTR and TrkA contributes to the downstream signaling [55], [56]. These findings indicate that p75NTR is essential to improve TrkA signaling activation. Moreover, p75NTR delays TrkA internalization and degradation upon NGF treatment and thereby facilitates the prolongation of cell surface TrkA signaling [57]. A previous study reported that TrkA alone was sufficient for NGF-induced neurite outgrowth in PC12 cells [58]. In contrast to that, our observations suggest that p75NTR plays a subtle but critical role in NGF-induced PC12 differentiation. This is consistent with a previous finding in which p75NTR has been shown to be essential for NGF-induced growth arrest by activating Akt in PC84 cells, a mutant p75NTR-deficient PC12 cell line [59]. It was found that PC84 cells continued to proliferate instead of differentiating in the presence of NGF. Intrabodies provide a valuable tool for future studies of this receptor at the posttranslational level. As the number of recombinant antibodies generated from large consortia aiming to cover entire proteome is rapidly increasing [13], [18], [60], their possible use to analyze their antigens by an intrabody strategy may significantly contribute to the functional analysis of proteins with yet unknown roles.

## Materials and Methods

### Cell cultures and transfections

Human embryonic kidney (HEK) 293T cells (ATCC) were cultured in Dulbecco modified Eagle medium (DMEM)/high glucose (4.5 g/L) and L-glutamine (2 mM) supplemented with 8% (v/v) fetal calf serum (FCS), and 1% (v/v) penicillin/streptomycin (PAA, Germany) and cultivated at 37°C in a 7% CO_2_ incubator. Rat pheochromocytoma PC12 cells (DSMZ) were cultured in RPMI 1640/L-glutamine (2 mM) supplemented with 10% horse serum (HS) and 5% (v/v) FCS (PAA, Germany). Mouse neuroblastoma x mouse spinal cord hybrid NSC19 cells (a kind gift from Prof. Brigitte M. Jockusch, Zoological Institute, Technische Universität Braunschweig, Germany) were maintained in DMEM/high glucose (4.5 g/L) and L-glutamine (2 mM) supplemented with 10% (v/v) FCS. PC12 and NSC19 cells were cultivated at 37°C in a 5% CO_2_ incubator. Cells were passaged in every 2–3 days in 1∶10 (for HEK 293T) or 1∶2 (for PC12 and NSC19) dilutions.

HEK293T cells were transfected by HEKFectin (Bio-Rad, Germany), while PC12 and NSC19 cells were transfected by NeuroMag (OzBiosciences, France) according to the supplier's instructions or by electroporation (Bio-Rad Gene Pulser Xcell™, Germany). For electroporation, 20 µg of endotoxin-free Midi-Prep prepared DNA was mixed with 2.4×10^6^ PC12 cells in 400 µL of RPMI 1640 medium and transferred into a 0.4 cm electrode cuvette (Bio-Rad, Germany). Single pulse (300 v, 950 µF) was applied at RT. After electroporation, cells were immediately replated with culture medium in poly-L-lysine pre-coated culture plates. Transfection efficiency was more than 70% as determined by flow cytometry after 24 hr.

### Plasmid construction

Expression vector for antigen production in HEK 293T cells:

The extracellular domain cDNA of mouse p75NTR was amplified from RZPD (German Science Centre for Genome Research, Germany) clone IRAVp968B1065D6 by PCR using the primer pair of 5′-TGC CAT GGC AAA GGA GAC ATG TTC CAC AGG CAT GT-3′ and 5′-AGA CGC GGC CGC AGG AAT GAG GTT GTC AGC GGT GCC T-3′. A mouse p75NTRex-Fc fusion construct was generated by cloning the PCR product via *Nco*I and *Not*I into pCMV-hIgG1-Fc-XP [16], a modified pCMV vector containing the human IgG1 Fc domain. The human antibody Vκ3 (hVκ3S) subfamily signal sequence and the mouse antibody heavy chain VH3 (mVH3S) subfamily signal sequence containing a synthetic intron proceeded secretion of the mouse p75NTRex-Fc fusion protein in HEK 293T cells. The resulting expression vector for mouse p75NTRex-Fc fusion protein was named pCMV-mp75NTRex-Fc.

Expression vectors for scFv productions in *E. coli*:

Primers 5′-GCC TAC GGC AGC CGC TGG-3′ and 5′-GAT CCT CTT CTG AGA TGA G-3′ were used to amplify the scFv cDNA sequences from the phage display vector by PCR. The *E. coli* expression vectors pOPE101-SH325-A11, pOPE101-SH325-B6, and pOPE101-SH325-G7 were generated by cloning the scFv cDNA sequences into *E. coli* expression vector pOPE101-XP [61] via *Nco*I/*Not*I.

Knockdown vectors:

The bicistronic knockdown vectors encode p75NTR-specific ER retained intrabodies and a farnesylated EGFP (EGFP-F) as a reporter gene directed by a modified EMCV-R IRES element (Fig. 1). The sequence containing His_6_-tag, ER retention sequence KDEL and cDNA of encephalomyocarditis virus (EMCV) internal ribosomal entry site (IRES) element [24] in order were subcloned into the knockdown vector via *Not*I/*Nco*I. The cDNA sequence of EGFP-F [6] were subcloned into the knockdown vector via *Nco*I/*Xba*I. Three p75NTR-specific scFvs were amplified by PCR using the reverse primer 5′-ATG TGC GGC CGC AGA GGA CGG T-3′ with three corresponding forward primers 5′-GGC CGC GCG CAC TCC GAG GTG CAG CTG TTG GAG ACC GGG GGA-3′, 5′-GGC CGC GCG CAC TCC CAG GTG CAG CTG GTG CAG TCT GGG GGA-3′, 5′-GGC CGC GCG CAC TCC GAG GTG CAG CTG GTG CAG TCT GGG GGA-3′. The DNA encoding the scFv against the hapten 2-phenyloxazoline-5-one (phOx) was amplified by PCR using 5′-ACA GGC GCG CAC TCC CAG GTG CAG CTG GTG CAG TCT-3′ and 5′-CCA GGA GTT CAG GTG CTG-3′. The resulting PCR products were digested with *BssH*II and *Not*I and ligated into the knockdown vectors.

### Antigen production for antibody phage display

One day before transfection, HEK293T cells were seeded onto a 10-cm petri dish using DMEM/high glucose supplemented with 1% (v/v) penicillin/streptomycin and 8% (v/v) FCS. HEK293T cells were transfected with the plasmid of pCMV-mp75NTRex-Fc. Twenty four hours after transfection, the medium was changed to DMEM/high glucose supplemented with 1% (v/v) penicillin/streptomycin and 4% (v/v) bovine low IgG level FCS (PAA, Germany). Expressed p75NTRex-Fc fusion protein was collected in the supernatant every 2 days for 14 days and purified by affinity chromatography using a HiTrap™ 1 mL protein A HP column on an ÄktaPrime system (GE Healthcare, Sweden) according to manufacturer's instructions.

### Selection of recombinant antibodies against the p75NTR extracellular domain

ScFvs against the p75NTR extracellular domain were selected from the naïve human antibody gene library HAL4 (Kappa) and HAL7 (Lambda) [16]. The library was packaged using Hyperphage [62], [63] to allow polyvalent display in the first panning round and mixed before panning. The panning and selection was performed as described previously [64]. The p75NTRex-Fc fusion protein was used panning antigen. Human IgG1 Fc (N Protein standard SL, DADE Behring, Germany) was used for preincubation of the library and for competition during the panning procedure to omit the selection of binders specific to the human IgG1 Fc domain.

### Production and purification of soluble p75NTR specific scFvs

Soluble p75NTR-specific scFvs were produced in shaking flasks using the *E. coli* strain XL1-Blue MRF' (Stratagene, Netherland) transfected with the pOPE101 constructs according to the procedure previously described [65]. Immobilized metal affinity chromatography (IMAC) was used for soluble scFv purifications from periplasmic extracts. Chromatography was performed using 0.5 mL Chelating Sepharose™ Fast Flow (GE Healthcare, Germany) according to the manufacturer's instruction.

### Antigen ELISA

A BD Falcon™ 96-well flexible plate (BD, USA) was coated with 100 ng of p75NTRex-Fc fusion protein or controls in 100 µL of PBS per well at 4°C overnight. The coated plate was blocked with 100 µL of FCS per well for 1.5 hr at 37°C and then washed 3× with PBST. The plate was then incubated with 250 ng of the p75NTR-specific scFvs for 1.5 hr at 37°C. After 3× washing with PBST, these scFvs were detected by anti myc-tag mAb (hybridoma, 1∶500) and goat anti-mouse IgG (Fab specific) antiserum conjugated with horseradish peroxidase (HRP) (1∶5,000, Sigma, USA). Binding was visualized with TMB (3,3′,5,5′-tetramethylbenzidine) substrate and stopped by 100 µL of 1 N sulphuric acid per well. The absorbance at 450 nm with subtracting the 620 nm reference was measured by a SUNRISE microtiter plate reader (Tecan, Germany).

### Competition ELISA

BD Falcon™ 96-well flexible plates were coated with 100 ng of the p75NTRex-Fc fusion protein for each well overnight at 4°C and blocked with FCS for 1.5 hr at 37°C. The p75NTR-specific scFvs were added in serial dilutions. After incubation for 1.5 hr at 37°C, the plates were washed 3× with PBST. The plates were again incubated with mouse anti-p75NTR mAb (MLR2, 1∶5,000, Abcam, UK) for 1.5 hr at 37°C and washed 3× with PBST. The scFvs and mouse anti-p75NTR mAb (MLR2) were detected by mouse anti-His_5_ mAb HRP conjugated (1∶5,000, Qiagen, Germany) or goat anti-mouse IgG (Fc specific) antiserum HRP conjugated (1∶5,000, Sigma, USA), respectively. Signals were developed and measured as described above.

### Surface plasmon resonance (SPR) spectroscopy

The binding kinetics of scFvs was measured by surface plasmon resonance spectroscopy using a Biacore2000 and a CM5 chip (Biacore, Sweden). The antigen p75NTRex-Fc and the recombinant rat TrkA/Fc chimera (rat TrkAex-Fc, R&D systems, USA) were separately coupled on to the chip via amine coupling according to the manufacturer's instructions. The binding experiment was performed at a flow rate of 30 µL/min using PBST (0.005% peroxidase-free Tween20) at room temperature (RT). Chip regenerations were done by 10 mM glycin buffers (pH1.5 or pH2.0). The p75NTR-specific scFvs with concentrations from 2.5 nM to 200 nM were injected with an association time of 5 min and a dissociation time of 10 min. Curve fittings were done using the model for 1∶1 binding with drifting baseline.

### Determination of intracellular expression of the p75NTR-specific scFvs

Harvested PC12 cells were centrifuged at 300× *g* for 5 min after 2× washing with pre-cold PBS. Appropriated amount of lysis buffer (1% Nonidet P-40, 10 mM Tris (pH 7.6), 150 mM NaCl, 5 mM EDTA, 1 mM PMSF, 5 µg/mL Aprotinin, 5 µg/mL Leupeptin, 5 µg/mL Pepstatin) was used to lyse cells. After incubated on ice for 30 min, the cells were centrifuged at 4°C, 13,400× *g* for 10 min. The supernatant was collected and stored at −20°C until analysis. After PAGE and blotting, the membrane was incubated with mouse anti-His_5_ mAb (1∶2,000, Qiagen, Germany) for 1 hr at RT and followed by 3× washing with PBST. The membrane was subsequently incubated with goat anti-mouse IgG antiserum (Fab-specific) alkaline phosphatase (AP) conjugated (1∶5,000, Sigma, USA) for 1 hr at RT. The bound antibodies were visualized by NBT/BCIP substrate after 3× washing with PBST.

### Detection of unfolded protein response (UPR)

Transiently transfected PC12 cells from different sampling intervals (2–8 days) were sorted by BD FACSAria™ II gated for the EGFP-F expression. Cell extracts were prepared from the sorted cells. After PAGE and blotting, the membrane was incubated with rabbit anti-GRP94 antiserum (1∶1,000, Sigma, USA) and rabbit anti-GAPDH antiserum (1∶5,000, Sigma, USA) for 1 hr at RT, separately. After 3× washing with PBST, the membrane was subsequently incubated with goat anti-rabbit IgG antiserum AP conjugated antibody (1∶5,000, Sigma, USA) for 1 hr at RT. The bound antibodies were visualized by NBT/BCIP substrate.

### Assay for binding to denatured p75NTR

250 ng of p75NTRex-mFc was denatured with 5× Laemmli buffer at 95°C for 10 min. After PAGE preparation and blotting, 1 µg of each p75NTR-specific scFv-Fc recombinant antibody was used to stain the membrane for 1.5 hr. The membrane was then incubated with goat anti-human IgG Fc antiserum AP conjugated antibody (1∶2,000, Dianova, Germany) for 1 hr at RT and followed by 3× washing with PBST. Binding antibodies were visualized as described above.

### P75NTR surface staining and flow cytometry

A total of 2×10^5^ cells PC12 or NSC19 cells were transferred into a FACS tube (Greiner, Germany). Cells were washed with FACS buffer (2% (v/v) FCS, 5 mM EDTA in PBS) and centrifuged at 300× g for 5 min at 4°C. The cell pellets were resuspended in 100 µL of the mouse anti-p75NTR mAb (MLR2, 1∶200) solution or 250 ng of p75NTR-specific scFvs in 100 µL volume, and then incubated for 1 hr on ice. The cell pellets stained by the p75NTR-specific scFvs were washed and subsequently resuspended with mouse anti His_6_-tag mAb (1∶100, Roche, Germany) in 100 µL volume and incubated for 30 min on ice. Afterwards, all pre-stained cells were washed and incubated in 100 µL of goat anti-mouse IgG (Fc_γ_ specific) F(ab′)2 fragment antiserum allophycocyanin (APC) conjugated (1∶200, Jackson ImmunoResearch, Germany) for 30 min on ice. After 2× washing, cells were resuspended in 500 µL of FACS buffer and kept on ice until analysis by flow cytometry using Beckman Coulter FC500. Data were analyzed by CXP analysis software.

### Real-time quantitative reverse transcription PCR (real-time qRT-PCR)

Total RNA was extracted from PC12 cells using RNeasy Mini Kit (Qiagen, USA) and first-strand cDNA was synthesized by SuperScript™ II reverse transcriptase with Oligo (dT)_12–18_ primer (ROTH, Germany) according to the manufacturer's instructions. The specific primers for Bcl-xL (forward: 5′- CGG CGG CTG GGA CAC TTT TG -3′ and reverse: 5′- CCG ACT GAA GAG TGA GCC CAG C -3′) and β-actin (forward: 5′- CGG TCA GGT CAT CAC TAT -3′ and reverse: 5′- TGT TGG CAT AGA GGT CTT -3′) were examined by Basic Local Alignment Search Tool (BLAST®). For real-time qRT-PCR, the reaction mixtures were prepared according to the manufacturer's instruction of SsoFast™ EvaGreen® Supermix (Bio-Rad, Germany) and the reactions were performed using CFX96™ Real-time PCR system (Bio-Rad, Germany). The reaction mixtures were pre-denatured at 95°C for 3 min, and then followed by 40 reaction cycles (95°C for 15 s; 60°C for 15 s; and 72°C for 30 s). To verify primer specificities, melting curve analyses were performed by increasing the temperature from 65°C to 95°C (0.5°C in every 5 s). The mRNA expression level of Bcl-xL in each sample was normalized to the β-actin mRNA expression level. The real-time qRT-PCR reactions were performed in triplicate.

### NGF-induced differentiation of PC12 cells and immunocytochemistry

PC 12 cells were transfected with appropriate intrabody constructs by electroporation as described previously and plated in poly-L-lysine coated 24-well plates in the concentration of 8×10^5^ cells per well. Two days after transfection, differentiation was induced by changing medium with 500 µL of fresh culture medium containing 200 ng/mL 2.5 s NGF (Chemicon, Germany). Medium was changed every 2 days. Six days after transfection, medium was aspirated from the wells and washed 2× with PBS. The cells were then fixed with 4% paraformaldehyde (PFA) at RT for 10 min and washed 3× with PBS for 5 min. The cells were stained with 100 ng/mL DAPI in PBS at RT for 5 min. Slides were then shortly washed with PBS for 3 times and mounted upside down on slides in Fluoro-gel (Electron Microscopy Sciences, Germany). The slides were preserved at 4°C until analysis with Zeiss Axiovert 200 fluorescent microscope (Zeiss, Germany).
